# Evaluation of a newer (1, 3)-β-D-glucan chemiluminescent immunoassay for invasive candidiasis: A study from a tertiary care center

**DOI:** 10.22034/cmm.2024.345199.1513

**Published:** 2024-05-10

**Authors:** Sudesh Gourav, Gagandeep Singh, Lokesh Kashyap, Bhaskar Rana, Swet Raj, Immaculata Xess

**Affiliations:** 1 Department of Microbiology, All India Institute of Medical Sciences, Ansari Nagar, New Delhi-110029, India; 2 Department of Anesthesiology, Pain Medicine & Critical Care, All India Institute of Medical Sciences, Ansari Nagar, New Delhi-110029, India

**Keywords:** (1, 3)-β-D-glucan, EORTC/MSGERC, FungiXpert®, Invasive candidiasis

## Abstract

**Background and Purpose:**

Invasive candidiasis (IC) in the hospitalized population is one of the leading causes of invasive fungal infections (IFIs). Microbiological diagnosis of IC suffers due to poor sensitivity of blood culture and relative inaccessibility to more sensitive modalities. (1, 3)-β-D-glucan (BDG) is a cell wall polysaccharide found in a range of fungi. Various commercial assays are available based on various detection techniques. This study aimed to assess the diagnostic performance of the FungiXpert® Fungus BDG Detection Kit by Genobio Pharmaceutical Co. Ltd. (Tianjin, China), based on chemiluminescent method, for diagnosis of candidemia and deep-seated candidiasis.

**Materials and Methods:**

In total, 80 patients (34 males and 46 females) were included with a median age of 35 years old. In accordance with EORTC/MSGERC definitions, 39 patients had proven IC. The number of patients within the probable, possible, and no IC (taken as control) groups were 8, 4, and 29, respectively. Blood samples were collected for fungal blood culture and BDG assay.

**Results:**

After exclusion of cases with evidence of concurrent IFI other than IC, median serum BDG was 0.63 ng/ml for proven IC; while it was 0.04 ng/ml for NO IC. Sensitivity, specificity, positive, and negative predictive values were 60.52%, 81.81%, 85.18%, and 54.54%, respectively. Positive likelihood ratio was 3.32. While the assay performed best for *Candida tropicalis* with median BDG of 1.92 ng/ml and sensitivity of 92.3%, its performance was worst for *Candida parapsilosis*, with median BDG of 0.04 ng/ml and sensitivity of 44.44%. Overall mortality rate was 65.62% in the BDG positive group, which was significantly higher than that in the BDG negative group (33.33%).

**Conclusion:**

The performance of the FungiXpert® Fungus BDG Detection Kit was acceptable for invasive candidiasis in the present resource-limited setup. The major advantages of this assay were the ease of performance in a semi-automated cartridge format, relatively lower cost per test, non-reliance on glucan-free procedures or instruments and minimal hands-on procedure.

## Introduction

Invasive fungal infections (IFIs) are an emerging problem worldwide with invasive candidiasis (IC) responsible for the majority of cases [ 1 ]. In a review of IFIs in intensive care units (ICUs), the incidence of candidemia was shown to vary between 3.5 and 16.5/1,000 admissions, depending on different studies and countries [ 2 ]. In a study on IFIs in Indian patients from a tertiary care center, overall prevalence was found to be 7.6%, with invasive candidiasis contributing to 20.9% of IFIs [ 3 ]. Commencing empiric treatment of candidemia more than 12 h after the first positive blood culture has been shown to be associated with a greater risk of mortality [ 4 ]. Therefore, rapid diagnostic tests are needed to avoid delays in appropriate antifungal therapy.

Sensitivity of antemortem blood cultures range from 21% to 71% in studies of autopsy-proven invasive candidiasis [ 5 ]. Blood cultures will not diagnose deep-seated candidiasis that is not associated with candidemia. Unfortunately, the sensitivity of deep-seated cultures is limited itself [ 6 ]. Components of *Candida* yeast cells that are remnants of prior candidemia or released from infected tissue sites can be detected by non-culture diagnostic tests to identify cases that are missed by blood cultures [ 5 ]. Despite these limitations, blood culture is considered the gold standard for diagnosis of IC [ 7 ].

New modalities for diagnosis are needed to complement cultures, in particular to identify around 50% of patients with blood culture-negative IC. Mannan antigen, anti-mannan antibody, and polymerase chain reaction can diagnose candidemia before blood cultures. They have shown promising results but are not widely available yet [ 8 - 10 ].

(1, 3)-β-D-glucan (BDG) is a cell wall polysaccharide molecule found in a wide variety of fungi, including *Candida* spp., *Aspergillus* spp., and *Pneumocystis jirovecii*; important exceptions among medically important fungi are Mucorales, *Cryptococcus* spp., and the yeast form of *Blastomyces* [ 11 ]. Factors associated with falsely elevated BDG concentration are colonization with *Candida* or mold, receipt of intravenous blood products or certain β-lactams, hemodialysis or hemofiltration, infection with some gram-positive bacteria, cellulose dressings, enteral nutrition, mucositis, and disruptions of gastrointestinal tract integrity [ 5 ]. Literature on the use of BDG for diagnosis of invasive candidiasis shows sensitivity ranging from 61% to 82%, while specificity ranged from 66% to 83%. In summary, it is unclear whether BDG testing helps the detection of infection at an early stage. It is also unclear whether a pre-emptive strategy (supported by BDG testing) leads to earlier diagnosis and better outcomes [ 12 ].

Various commercial assays for the detection of BDG are available around the globe based on various detection techniques. They are based on the ability of the BDG molecule to induce formation of clot in the hemolymph of horseshoe crabs. An overview of major commercially available BDG assays has been included in the following paragraphs.

Fungitell (Associates of Cape Code, Falmouth, MA, USA) was the first FDA-cleared and CE marked BDG assay. It uses a chromogenic method for BDG detection. In a meta-analysis restricted to only 10 low-bias studies, the sensitivity and specificity were found to be 80% and 63%, respectively [ 13 ]. There was substantial heterogeneity between studies.

Fungitell STAT (Associates of Cape Code, Falmouth, MA, USA) is a design modification of the Fungitell assay. It was developed to address the need for a single use test format and smaller kit size in comparison to the 96-well plate format of the Fungitell assay. In a study to establish categorical agreement between Fungitell and Fungitell STAT, positive percent agreement values with and without indeterminate results were 74% and 99%, respectively. Negative percent agreement values were 91% and 98% with and without indeterminate results, respectively [ 14 ].

For the Wako β-Glucan assay (Wako Pure Chemical Industries, Osaka, Japan) based on turbidometric method, a comparative analysis with the Fungitell assay was performed. Sensitivity, specificity, and positive and negative predictive values for candidemia were 86.7%, 85.0%, 6.0%, and 99.8% in the Fungitell assay and 42.5%, 98.0%, 19.0%, and 99.4% in the Wako assay, respectively [ 15 ].

For the Dynamiker Fungus BDG assay (Dynamiker Biotechnology Ltd, Tianjin, China) based on spectrophotometric method, one study found the sensitivity, specificity, and diagnostic odds ratio for proven/probable IFI to be 81.4%, 78.1%, and 15.5, respectively. It showed fair agreement with the Fungitell assay [ 16 ].

This research aimed to present the evaluation of FungiXpert® BDG assay by Genobio Pharmaceutical Co. Ltd. (Tianjin, China), based on chemiluminescent method, for diagnosis of candidemia and deep-seated candidiasis.

## Materials and Methods

### Study population

This prospective case control study was conducted on suspected cases of invasive candidiasis. It included 80 critically ill (defined as requiring intensive care for functioning of vital organs) patients admitted to various wards (with critical care support) and ICUs of All India Institute of Medical Sciences, New Delhifrom July 2022 to February 2023. All microbiological investigations were performed on samples sent for routine clinical workup.

Demographic details of all patients were recorded. Associated host risk factors, and clinical, microbiological and radiological findings were analyzed and patients were categorized as proven, probable, possible, or no IC according to EORTC/MSGERC Definitions of Invasive Fungal Diseases: Summary of Activities of the ICU Working Group [ 17 ]. 

In brief, patients with positive blood culture for *Candida* spp. were categorized as proven IC; patients with ≥ 1 host factor, ≥ 1 clinical criterion, and two consecutive BDG positive results were categorized as probable IC; patients with ≥ 1 host factor, ≥ 1 clinical criterion, and negative microbiological investigations were categorized as possible IC; and patients fitting into none of the above-mentioned groups were categorized as no IC (taken as control). Host factors and clinical criteria were defined according to EORTC/MSGERC definitions.

In this study, 3 ml of blood sample was taken and serum was separated for BDG assay. At least two samples were collected from each patient with at least a 24-h interval between them. Moreover, 8-10 ml of blood for fungal blood culture was collected from a peripheral site in BD BACTEC (Becton Dickinson, NJ, USA) Mycosis IC/F bottles. At least two blood cultures were collected within 24 h from each patient.

The study was approved by the institutional ethical committee of All India Institute of Medical Sciences, New Delhi (Ref no. IEC-3/14.01.2022, RP-20/2022). Consent was not needed as the results were derived from samples sent for routine clinical workup.

### Mycological examination

Blood cultures collected in BD BACTEC Mycosis IC/F bottles were incubated at 37°C for at least 7 days in the BD BACTEC Automated Blood Culture System (Becton Dickinson, NJ, USA). Gram stain was made and subculture on Sabouraud dextrose agar (HiMedia Laboratories, Mumbai, India) with antibiotics was done for bottles that flagged positive. Identification of the different *Candida* spp. was performed both by conventional methods (HiCrome *Candida* Differential Agar and HiMedia Corn Meal Agar, HiMedia Laboratories, Mumbai, India) and proteomic analysis using matrix assisted laser desorption ionization-time of flight mass spectrometry (VITEK MS system, BioMérieux, Marcy-I’Etoile, France).

### (1, 3)-β-D-glucan measurement

The BDG assay was run for all of the above-mentioned samples using the FungiXpert® Fungus BDG Detection Kit (Genobio Pharmaceutical Co. Ltd., Tianjin, China) as recommended by the manufacturer. Accordingly, 300 μL of serum sample was added to the reaction cup of the reagent strip and thoroughly mixed. It was incubated in the Full-Automatic Chemiluminescence Immunoassay System (FACIS-I, Genobio Pharmaceutical Co. Ltd., Tianjin, China). All of the subsequent steps were performed in an automated manner in the FACIS-I system. Briefly, BDG molecule in the sample was allowed to form an immune complex of magnetic bead coated with anti-BDG antibody- BDG antigen- horseradish peroxidase-labeled anti-BDG antibody.

After the completion of reaction, the precipitation complex was repeatedly washed by the action of a magnetic field. After addition of the chromogenic solution, the illuminance intensity value after the chemiluminescence reaction was read by the FACIS-I system. The concentration of BDG in the sample was calculated according to the standard curve. All the aforementioned reactions were performed inside the FungiXpert® reagent strip. Total duration of the assay was 60 min. The BDG concentration of ≥ 0.1 ng/ml was considered positive, ≤0.06 ng/ml was considered negative, and a value between them was considered indeterminate as per manufacturer recommendation.

All BDG results were recorded. Only patients with two consecutive positive BDG results were considered positive for BDG assay.

### Statistical analysis

Continuous variables have been described as median, range, and interquartile range (IQR: 25^th^ percentile to 75^th^ percentile). Categorical variables have been described as percentages with 95% confidence intervals.

Potential inter-group differences have been assessed using the χ2 test (for categorical variables) or Mann-Whitney test (for two group comparisons of quantitative variables). A receiver operator characteristic (ROC) curve was plotted to determine the ability of the serum BDG level to discriminate proven IC cases from cases with no IC. Sensitivity and specificity were determined to calculate the optimal BDG cut-off. The *P* values of less than 0.05 were considered statistically significant.

## Results

### Study population

In total, 80 patients (34 males and 46 females) were included in this study. The median age of the participants was 35 years old (range: 12-73 years). Most patients had underlying risk factors for IC. The proportion of patients with different underlying host factors was not analyzed formally as data was lacking for some of them. In accordance with EORTC/MSGERC definitions, 39 patients had proven IC. The number of patients within the probable, possible, and no IC groups were 8, 4, and 29 respectively. It is noteworthy that 38 patients were recruited from ICUs while 42 patients were from different wards. Characteristics of the different groups are summarized in Table 1.

**Table 1:** Characteristics of different study groups. Patients were categorized as proven, probable, possible, or no invasive candidiasis according to EORTC/MSGERC definitions of invasive fungal diseases: summary of activities of the intensive care unit working group

EORTC/ MSGERC group		Proven IC	Probable IC	Possible IC	No IC
Total Patients		39	08	04	29
Male/Female		14/ 25	06/ 02	02/ 02	12/ 17
Ward/ICU		26/ 13	01/ 07	01/ 03	14/ 15
Median Age		35	64	51	35
BDG Positive		23	08	–	09
BDG Negative		16	–	04	20
Median BDG (ng/ml)		0.55	8.05	–	0.05
Deaths	BDG+	15	06	–	06
	BDG-	08	–	02	04

BDG: (1, 3)-β-D-glucan, ICU: Intensive Care Unit, IC: invasive candidiasis

### Serum (1, 3)-β-D-glucan levels

Serum BDG assay was positive in 23 out of 39 (58.97%) cases with PROVEN IC; while it was positive in 9 out of 29 (31.03%) cases with NO IC (*P*=0.02). One patient in the PROVEN IC group, two patients in the PROBABLE IC group and seven patients in the NO IC group had evidence of concurrent IFI other than IC based on direct microscopy with/without culture (i.e. *Aspergillus* spp., *Pneumocystis jirovecii*, and other fungi). 

After exclusion of these cases to minimize bias, serum BDG assay was positive in 23 out of 38 (60.52%) cases with PROVEN IC (median: 0.63 ng/ml, range: 0.0215- 9.2333, IQR: 0.04- 4.06); while it was positive in 4 out of 22 (18.18%) cases with NO IC (median: 0.04 ng/ml, range: 0.0239- 8.3316, IQR: 0.03–1) (*P*=0.003). Sensitivity, specificity, positive predictive value, negative predictive value, and test accuracy were 60.52% (95% CI: 43–75), 81.81% (95% CI: 58–94), 85.18% (95% CI: 65–95), 54.54% (95% CI: 36–71), and 68.33%, respectively. 

**Figure 1:**
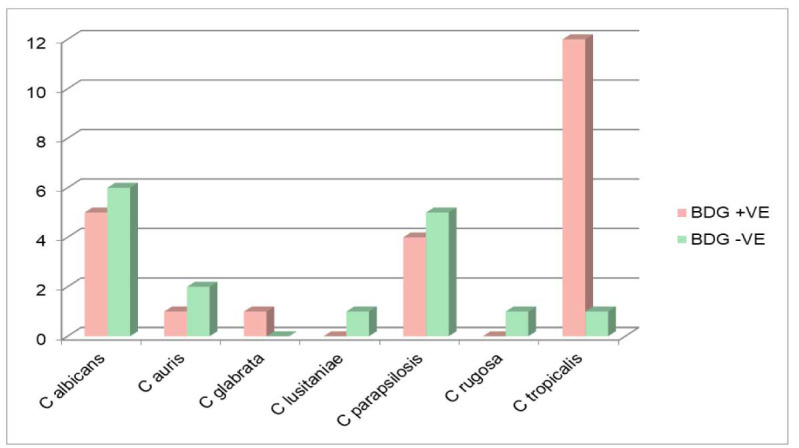
Number of cases in (1, 3)-β-D-glucan (BDG) positive and BDG negative groups among blood culture isolates of different *Candida* spp.

Moreover, positive likelihood ratio was 3.32. All subsequent statistical analyses were performed based on these values as standard for comparison. Among the 39 proven cases, *Candida albicans* was isolated from 11 cases (BDG was positive in 5/11 cases, median: 0.40 ng/ml, range: 0.0396-8.1987, IQR: 0.04-6.13), *Candida auris* from 3 cases (BDG positive in 1/3 cases, median: 0.05 ng/ml, range: 0.0327-1.6285, IQR: 0.04-0.84); *Candida glabrata* from 1 case (BDG positive in 1/1 case, BDG: 0.16 ng/ml); *Candida lusitaniae* from 1 case (BDG positive in 0/1 cases, BDG: 0.03 ng/ml); *Candida parapsilosis* from 9 cases (BDG positive in 4/9 cases, median: 0.04 ng/ml, range: 0.0215-8.01, IQR: 0.02-2.12); *Candida rugosa* from 1 case (BDG: positive in 0/1 case, BDG: 0.03 ng/ml), and *Candida tropicalis* from 13 cases (BDG positive in 12/13 cases, median: 1.92 ng/ml, range: 0.0507-9.2333, IQR: 0.55-8.05) (*P* values: 0.121, 0.504, 0.217, 1, 0.185, 1, and <0.001 respectively vs. control) (Figure 1).

The proven IC cases and cases with evidence of other IFI were excluded to minimize bias, and different factors with possible association with false positive BDG results were analyzed. Treatment-related factors (recent hemodialysis, use of cellulose filters, gauze packing, or use of intravenous immunoglobulin or albumin), use of various antimicrobials, and invasive bacterial infections were not found to be significant contributors for false positivity of BDG (Supplementary material 1).

Among patients with positive BDG assay, 15 out of 23 (65.21%) patients expired in the PROVEN IC group and 6 out of 9 (66.66%) patients expired in the NO IC group. Similarly, among patients with negative BDG assay, 8 out of 16 (50%) patients expired in the PROVEN IC group and 4 out of 20 (20%) patients expired in the NO IC group. Overall, 21 out of 32 (65.62%) patients expired in the positive BDG group and 12 out of 36 (33.33%) patients expired in the negative BDG group (*P*=0.014). Moreover, mortality was significantly higher in the positive BDG group even without a proven diagnosis of IC (*P*=0.032, Table 1).

**Figure 2:**
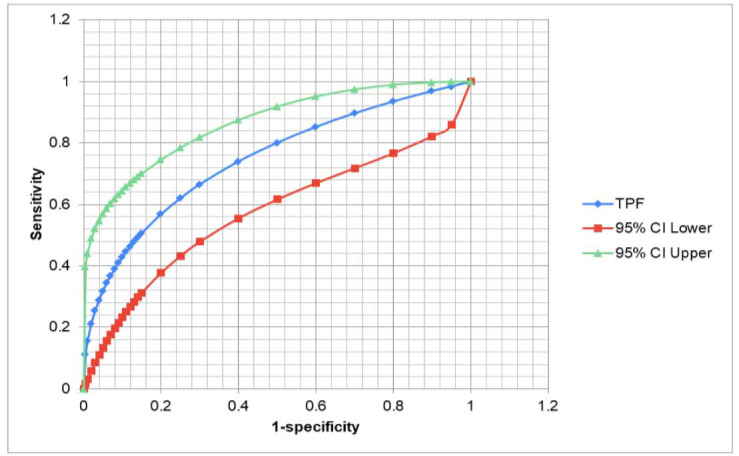
Receiver operating characteristic (ROC) curve for serum (1,3)-β-D-glucan (BDG). Cut-off between 0.06 ng/ml and 0.12 ng/ml gave the best compromise between sensitivity and specificity. Area under the curve (AUC) was calculated to be 0.746. TPF: true positive fraction

Sensitivity and specificity were determined for different cut-off values for determining a positive result (Table 2). A ROC curve was plotted taking 100-specificity and sensitivity in x-axis and y-axis, respectively (Figure 2).

**Table 2:** Properties of the FungiXpert® Fungus (1, 3)-β-D-glucan Detection Kit at different cut-off values

Cut-off	0.02 ng/ml	0.04 ng/ml	0.06 ng/ml	0.08 ng/ml	0.1 ng/ml	0.12 ng/ml	0.14 ng/ml	0.16 ng/ml
**Sensitivity (%)**	100	81.57	60.52	60.52	60.52	60.52	57.89	57.89
**Specificity (%)**	0	45.45	81.81	81.81	81.81	81.81	81.81	81.81

DiscussionInvasive fungal infections remain a significant problem in critically ill patients, with invasive candidiasis contributing to a majority of those infections. Up to 40- 50% of all episodes of candidemia may be associated with intravenous catheters, a common predisposing factor in these patients. Although there remains a possibility of overestimation due to possible inclusion of some cases of catheter colonization, differentiating colonization from true candidemia is difficult due to the poor sensitivity of blood culture [ 17 ]. In these scenarios, presumptive diagnosis of candidemia in critically ill patients with signs and symptoms of systemic infection is usually made by the use of risk prediction models or non-culture-based diagnostic assays [ 18 ]. Serum BDG detection is one of these non-culture-based methods.

In this study, the FungiXpert® Fungus BDG Detection Kit was evaluated for the diagnosis of candidemia and deep-seated candidiasis. After exclusion of cases with evidence of concurrent IFI other than IC, sensitivity, specificity, positive predictive value, negative predictive value, and test accuracy values were 60.52%, 81.81%, 85.18%, 54.54%, and 68.33% respectively. Moreover, positive likelihood ratio was 3.32.

While the assay performed best for *C. tropicalis*, it was acceptable for *Candida albicans*. Among the major agents, the assay performance was worst for *C. parapsilosis*. To emphasize the significance, these are the three most common agents of IC in our center (All India Institute of Medical Sciences, New Delhi). The assay was unable to detect one case of each *C. lusitaniae* and *C. rugosa*, and 2/3rd of the cases of *C. auris*. This showed the limitations of the assay in detection of different non-albicans *Candida* spp., as well as a possible explanation for inter-centre variability of test performance. 

Del Bono et al. reported similar variability previously, including lower sensitivity for *C. parapsilosis* [ 19 ].

Possible contribution of treatment-related factors, use of various antimicrobials, particularly beta-lactams, and presence of invasive bacterial infection were analyzed. None of them were shown to be contributing significantly to a positive BDG result. Overall, mortality was 65.62% in the BDG-positive group, significantly higher than 33.33% in the BDG-negative group.

As discussed in the introduction section, for the other commercially available BDG assays, large scale studies and meta-analyses have shown sensitivity and specificity within the range of 60- 80% for most of them. Major disadvantages of the relatively widely available assays (Fungitell, Fungitell STAT, and Wako β-Glucan assays) include higher cost per test, requirement of skilled laboratory staff to perform the test, and need for glucan-free collection system [ 14 , 15 ]. The FungiXpert® Fungus BDG Detection Kit performed similarly, with added advantage of relatively lower cost, non-reliance on special collection systems, and minimal hands-on work.

## Conclusion

Performance of the FungiXpert® Fungus BDG Detection Kit (Genobio Pharmaceutical Co. Ltd., Tianjin, China) was acceptable for invasive candidiasis in our setting. The major advantage of using this assay was the significantly lower cost per test, compared to other globally available assays. 

Unlike other assays, this assay does not require any glucan-free collection vials and other consumables, which reduces chances of false positivity. The ease of performance in a semi-automated cartridge format with minimal hands-on procedure is also helpful, as no special training or skill-sets are required and it can be performed by any technical staff. 

Relatively small sample size and absence of systematic assessment for *Candida* colonization were the major lacunae of this single-center study. Reliance on provided history by physicians for categorization of patients into probable, possible, or no IC groups was also a potential conflicting factor. The authors could not verify the causes of false positive BDG assay results with any better test available. This assay, which potentially provides a promising alternative in resource-limited settings requires further larger scale evaluation.
